# *Clostridium difficile* in Ground Meat, France

**DOI:** 10.3201/eid1604.091138

**Published:** 2010-04

**Authors:** Sylvie Bouttier, Marie-Claude Barc, Benjamin Felix, Sylvie Lambert, Anne Collignon, Frédéric Barbut

**Affiliations:** Université Paris-Sud 11, Châtenay-Malabry, France (S. Bouttier, M.-C. Barc, S. Lambert, A. Collignon); Hôpital Saint-Antoine, Paris, France (B. Félix, F. Barbut)

**Keywords:** Bacteria, Clostridium difficile, enteric diseases, ground meat, France, foodborne illnesses, letter

**To the Editor:** *Clostridium difficile* is a toxigenic enteropathogen responsible for 15%–20% of antimicrobial drug–associated diarrhea and for almost all cases of pseudomembranous colitis. Two protein toxins (TcdA and TcdB) play a major role in the pathogenesis of infections. *C. difficile* is also recognized as a cause of disease in several animal species, which could be potential reservoirs (*1*). In the past few years, the presence of *C. difficile* in raw diets for dogs and cats and in retail meat sold for human consumption has been reported in the United States and Canada at rates from 6% to 42% (*2*–*5*). To determine *C. difficile* contamination of meat in France, we evaluated 105 packages of ground beef (vacuum packed or not), 59 pork sausages, and 12 packages of feline raw diet meat purchased from 20 urban and suburban Paris retail stores and supermarkets during September 2007–July 2008.

*C. difficile* spores or vegetative forms in samples were found as described by Rodriguez-Palacios et al. (*4*). Briefly, 5 g of each sample was cultured in 100 mL of prereduced brain–heart infusion (BHI) broth supplemented with cefoxitin (10 µg/mL), cycloserine (250 µg/mL), and taurocholate (0.1%). After the samples were incubated under anaerobic conditions at 37°C for 72 h, subculturing with and without alcohol shock for spore selection was performed. The BHI broth culture was treated with 2 mL of absolute ethanol (1:1 vol/vol) for 30 min and centrifuged at 3,800 × *g* for 10 min, and the pellet was resuspended in 200 µL of prereduced BHI broth. Serial dilutions of the BHI broth and the pellet were injected onto Columbia cysteine agar supplemented with cefoxitin-cycloserine, taurocholate, and 5% horse blood and incubated anaerobically for 48 h at 37°C.

*C. difficile* colonies were identified classically, and susceptibilities to moxifloxacin, teicoplanin, vancomycin, metronidazole, linezolid, levofloxacin, telithromycin, erythromycin, and lincomycin were determined by the agar disk-diffusion methods described by the French Society for Microbiology (www.sfm.asso.fr). PCR amplifications of a species-specific internal fragment of the triose phosphate isomerase (*tpi)* gene, an internal fragment of the toxin B (*tcdB*) gene, and the 3′ region of the toxin A (*tcdA*) gene were performed as described by Lemee et al. (*6*). Strains were characterized by toxinotyping according to Rupnik et al. (*7*) and PCR-ribotyping as described by Bidet et al. (*8*).

The detection threshold of the enrichment method was established by spiking known uninfected samples (ground beef, pork sausage, and feline raw diets) with vegetative cells and spores of *C. difficile* (VPI 10463 strain). For ground beef samples, the detection thresholds for vegetative forms and spores were 2 CFU/5 g and 4.5 CFU/5 g of meat, respectively. For pork sausages, the detection thresholds were 14 CFU/5 g and 38 CFU/5 g of sample after 72 h, for vegetative forms and spores, respectively. For feline raw diets, the detection threshold of spores was 2 CFU/5 g of sample. In addition, toxin B was detected in the culture supernatants by the cytotoxicity assay onto MRC-5 cells. Toxin detection showed 100% agreement with the culture method.

*C. difficile* was not detected in pork sausages or in commercial feline raw diets. *C. difficile* was isolated from 2 (1.9%) of 105 ground beef, but only from those packages that were vacuum packed. The anaerobic atmosphere of vacuum packaging could facilitate the survival of *C. difficile* and the germination of spores. These 2 isolates were fully susceptible to moxifloxacin, teicoplanin, vancomycin, metronidazole, and linezolid but resistant to levofloxacin, telithromycin, erythromycin, and lincomycin. They harbored genes encoding for Tpi protein and for TcdA and TcdB. The 2 strains belonged to the toxinotype 0 and PCR-ribotype 012. Toxinotype 0 was already identified in meat samples in Canada (*4*). PCR-ribotype 012 belongs to the 10 ribotypes most frequently isolated from humans (*9*).

The prevalence of *C. difficile* in ground meat in France is low compared with the prevalence reported by other countries. In Canada, Rodriguez-Palacios et al. (*4*) studied 60 beef samples and found the prevalence of *C. difficile* to be 20%. These same authors, by using a broader sampling scheme (214 meat samples), isolated *C. difficile* from 6% of the samples (*5*). Also in Canada, Weese et al. (*10*) reported that 12% of ground beef and ground pork samples were contaminated. In the United States, *C. difficile* was isolated from 42% of meat samples (beef, pork, and turkey products) (*3*).

For a better understanding of the sources of *C. difficil*e in France, it would be interesting to determine its prevalence in different animal fecal samples and the toxinotypes associated with animals. The low prevalence in retail meat in France could result from hazard analysis critical control point principles and microbiologic quality controls implemented throughout the food production chain, which help reduce the spread of *C. difficile* and minimize the risk for infection for the consumer.
